# Loss of health related quality of life following low-trauma fractures in the elderly

**DOI:** 10.1186/s12877-016-0259-5

**Published:** 2016-04-19

**Authors:** Jean-Eric Tarride, Natasha Burke, William D. Leslie, Suzanne N. Morin, Jonathan D. Adachi, Alexandra Papaioannou, Louis Bessette, Jacques P. Brown, Louisa Pericleous, Sergei Muratov, Robert B. Hopkins

**Affiliations:** Programs for Assessment of Technology in Health (PATH), St. Joseph’s Healthcare Hamilton, 25 Main Street West, Suite 2000, Hamilton, ON L8P 1H1 Canada; Department of Clinical Epidemiology and Biostatistics, McMaster University, Hamilton, Ontario Canada; Department of Medicine, University of Manitoba, Winnipeg, Manitoba Canada; Department of Medicine, McGill University, Montréal, Quebec Canada; Department of Medicine, McMaster University, Hamilton, Ontario Canada; Department of Medicine, Laval University, Quebec City, Quebec Canada; Amgen Canada Inc., Mississauga, Ontario Canada

**Keywords:** Frail elderly, Fractures, Health-related quality of life

## Abstract

**Background:**

To estimate the long-term change in health related quality of life (HRQoL) following low-trauma fractures among individuals receiving home care (HC) services or living in long-term care (LTC) facilities using linked healthcare administrative data from Ontario, Canada.

**Methods:**

HRQoL was estimated using the Health Utility Index (HUI-2) with the InterRai Minimum Data Set (MDS), a mandatory questionnaire for LTC and HC in the province of Ontario (population 14 million). The HUI-2, a validated HRQoL instrument, allows the calculation of health utility where 0 represents death and 1 the best imaginable health state. For reference, the HUI-2 utility value for Canadians aged 80–84 years is 0.61 and the minimal clinically important difference is 0.03. The MDS was linked to Ontario acute care databases for fiscal years 2007–2011 to identify low-trauma fractures using ICD-10-CA codes. Regression models were used to identify predictors of change in HRQoL from pre-fracture levels to 3 years post fracture for several populations. Low-trauma fractures included hip, humerus, vertebral, wrist, multiple and other.

**Results:**

Twenty-three thousand six-hundred fifty-five unique patients with low-trauma fractures were identified with pre- and post-fracture HRQoL assessments, of which 5057 individuals had at least 3 years of follow-up. Compared to patients receiving HC services (*N* = 3303), individuals residing in LTC (*N* = 1754) were older, taking more medications, and had more comorbidities. LTC patients had more hip fractures (49 % of total versus 29 %). For all fracture types, HRQoL decreased immediately following fracture. Although levels rebounded after the first month, HRQoL up to 36 months never returned to pre-fracture levels even for non-hip fracture. For both HC and LTC cohorts, clinically important and statistically significant decreases in HUI-2 utility scores were observed 36 months post fracture. Of the 6 HUI-2 domains, mobility had the largest impact on change in HRQoL. Regression analysis indicated that living with a musculoskeletal disorder or a neurological condition and living in LTC were associated with greater decrements in utility following a fracture.

**Conclusions:**

Based on the analysis of one of the largest studies on HRQoL to date, among individuals living in LTC facilities or receiving HC services, fractures have a significant permanent impact on HRQoL up to 3 years following fracture.

**Electronic supplementary material:**

The online version of this article (doi:10.1186/s12877-016-0259-5) contains supplementary material, which is available to authorized users.

## Background

Fragility fractures are a result of progressive decrease in bone density and bone strength [1]. This presents a public health issue and has recently become a major focus of disease prevention activities for clinicians [2, 3]. Fragility fractures are associated with a higher risk of subsequent fractures (“fracture cascade”) and increased mortality [2, 4]. Although hip, vertebral, upper arm and forearm have been long considered the most common fracture sites, the epidemiology of the condition appears to be changing: non-hip, non-vertebral fractures now account for a substantial portion, up to 40 %, of fragility fractures, representing a non-negligible economic burden [5, 6].

Fracture-related disability has an enormous impact on patients’ quality of life. Measured by a number of validated tools, health related quality of life (HRQoL) has been shown to consistently decrease following fragility fractures [7–10]. Few studies, however, have assessed changes in HRQoL over the long term. The results of those that did demonstrated a lasting negative impact on HRQoL of hip and spine fractures in particular, and a rebound in HRQoL to pre-fracture levels in sites such as forearm or ankle [11–13].

Although very informative, existing studies have important limitations. They rarely focus on the elderly population receiving HC services or residing in LTC facilities, instead focusing on all patients, although the demographic transformation in developed countries prompts researchers to explore health aspects of this population cohort in more detail [14]. Institutionalized patients, who are hypothesized to suffer more pronounced HRQoL losses after fractures, may be underrepresented in these studies due to recruitment bias in research conducted in community-based settings [15]. Also, the relatively small sample sizes often limits interpretation of their results [13, 16–18]. The objective of this study was therefore to estimate the long-term change in HRQoL following fractures among the elderly receiving home care (HC) services or living in long-term care (LTC) facilities using linked administrative databases from Ontario, Canada.

## Methods

### Study overview

Several administrative datasets from the Canadian province of Ontario (population 14 million) were linked to document post-fracture changes in HRQoL among individuals receiving HC services or living in LTC. HRQoL, as evaluated with the Health Utility Index-version 2 (HUI-2) instrument, was compared 3 years post fracture with pre-fracture levels according to fracture site and residency status (LTC or HC).

### Study population

In a first step, Ontarians aged 50 years and older with any acute care admission, emergency visit or same day surgery due to a fracture during fiscal years (FY) April 1, 2006 to March 31, 2011 (FY 2007/11) were identified using two databases hosted by the Canadian Institute for Health Information (CIHI): 1) Discharge Abstract Database (DAD) [19] for acute care admissions; and 2) National Ambulatory Care Reporting System (NACRS) [20] for emergency visits and same day surgery visits. Fracture sites were identified using the International Classification of Diseases (ICD-10-CA) fracture codes: hip, humerus, clinical vertebral, wrist (distal forearm), other sites (femur, lower leg including tibia, fibula knee and foot, lower arm including radius and ulna, ribs, shoulder, arm, sternum, clavicle, pelvis) and multiple fractures (i.e. more than 1 of the above). Fractures with high-impact trauma codes were excluded. The ICD-10-CA codes used to identify low-trauma fractures are provided in Additional file 1.

In a second step, the DAD and NACRS records for those individuals identified were linked to the Home Care Reporting System (HCRS) [21] and the Continuing Care Reporting System (CCRS) [22] to identify those individuals who received HC services or were living in LTC facilities during FY 2007/11, respectively. To be included in the analyses, individuals had to have pre- and 36-month post-fracture HRQoL measurements in either setting (i.e. LTC or HC). This means that individuals living at home who transferred to LTC or HC following a fracture were not included in our analysis due to the lack of HRQoL data pre fracture (see more details in next section). To document the long-term impact of fracture on HRQoL, individuals had to have at least 3 years of follow-up after a fracture. Two analytic cohorts of Ontarians experiencing a fracture were created depending on their residency status (living at home but receiving HC services or living in a LTC facility).

### Health-related quality of life measurement

The primary outcome of this study was the change in HRQoL 3 years following a fracture. HRQoL was estimated using the HUI-2 which is included among other instruments/questions in the InterRai Minimum Data Set (MDS) [23]. The MDS is a mandatory questionnaire for all Ontarians living in publicly-funded LTC facilities or for patients who receive nurse-based HC services in Ontario as prescribed by a physician. In contrast with CIHI databases which are triggered by an admission and are purely administrative (e.g. no HRQoL information available for analysis), the MDS is administered to every patient by a nurse or social worker at a minimum quarterly, or with any change in clinical condition. In addition to the HUI-2 instrument, the MDS includes more than 200 questions on clinical outcomes and resource utilization, for both individuals receiving HC services or living in LTC.

The HUI-2 is a validated instrument to measure HRQoL and it has been used in hundreds of studies and clinical settings [24]. The HUI-2 measures HRQoL in six dimensions: sensation, mobility, emotion, cognition, self-care and pain. Each domain has 3–6 levels of ability/disability and the responder has to select one level for each domain. Using a scoring algorithm [25], the individual responses to each of the 6 domains are transformed into a utility score where 0 represents death and 1 the best imaginable health state [24]. As a reference, on a 0 (death) to 1 (full health) scale, the HUI-2 utility score was 0.61 for Canadians aged 80–84 years and living in the community [26]. The minimal clinically important difference (MCID) in the HUI-2 utility score is 0.03 while the MCID associated with each of the 6 domains of the HUI-2 is 0.05 [24]. In our study, the HUI-2 utility score was calculated for the 1-year period preceding the fracture and at post-facture periods of 1, 3, 6 months, and every 6 months thereafter for up to 3 years. Changes in HUI-2 utility scores compared to pre-fracture level, also known as disutilities, were calculated 36 months post fracture to document the impact of fracture in HRQoL.

### Statistical analyses

Continuous variables (e.g. age) were summarized using mean values and standard deviations (SDs) while discrete variables (e.g. presence of comorbidities) were represented using percentages. Student t-tests and chi-square tests were used to compare study population characteristics or fracture types between the LTC and HC cohorts.

Paired t-tests were used to compare assessments across time for the HUI-2. In addition, regression analyses were conducted to explain the disutility associated with fracture at 3 years post fracture (i.e. 3-year HUI-2 utility score minus pre-fracture HUI-2 utility score). Covariates included age, sex, fracture type, residency status (HC or LTC), and comorbidities. Comorbidities were derived from the MDS questionnaire and were represented in the analyses as a binary variable for seven categories: endocrine/metabolism (diabetes, hypo/hyper-thyroidism), heart/circulation (arrhythmia, congestive heart failure, hypo/hyper-tension, peripheral vascular disease), musculoskeletal (arthritis, amputation, osteoporosis, history of fracture), neurological (Alzheimer’s Disease, dementia, stroke, Parkinson’s Disease), psychiatric/mood (anxiety disorder, depression, bipolar disorder, schizophrenia), pulmonary (asthma, emphysema, chronic obstructive pulmonary disease), and sensory (cataracts, retinopathy, glaucoma, macular degeneration). In these analyses, generalized mixed effects models were used to account for skewness and heterogeneity. In addition to analyzing the combined HC and LTC cohorts, regression analyses were also conducted for the following four populations: 1) HC cohort only; 2) LTC cohort only; 3) individuals with neurological conditions; and 4) individuals without neurological conditions. To explore the impact of censoring in the data, an analysis was conducted to compare the decline in HRQoL following a fracture between those with 3 years of data and those who died before 3 years. We also explored the decline in HRQoL for different types of hip fractures including neck of the femur (ICD-10-CA S72.0), pertrochanteric (ICD-10-CA S72.1) and subtrochanteric (ICD-10-CA S72.2) fractures.

## Results

### Study population description

**Fig. 1:**
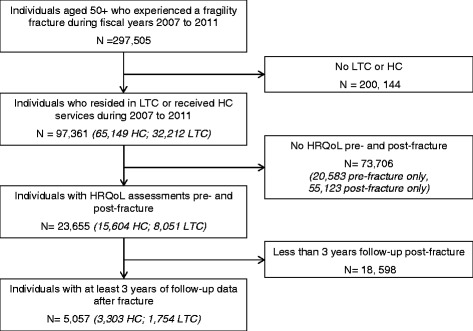
Flow Chart of Identification of Study Population

For FY 2007/11, 297505 unique Ontarians aged 50 years and older experienced a low-trauma fracture, of whom 33 % required either HC services (*N* = 65149) or were living in LTC (*N* = 32212). Of the patients that required HC or LTC, 21 % had pre-fracture assessments (i.e. MDS questionnaire was administered at least once during the 1 year preceding the fracture) and no post-fracture assessments, and 55 % had post-fracture assessments without pre-fracture assessments. The remaining 24 % (*N* = 23655) had both pre- and post-fracture assessments. Of those, 5057 unique individuals had 3 years of follow-up data post fracture, of who almost one third were living in LTC facilities (See Fig. 1).

Table 1 presents the baseline demographics of our study population for the two cohorts. Compared to patients receiving HC services (*N* = 3303), individuals residing in LTC (*N* = 1754) were older (84.6 versus 80.6 years, *p* < 0.001), were taking more medications (8.7 versus 7.3, *p* < 0.001), and had more comorbidities (8.0 versus 2.4, *p* < 0.001). For example, 88 and 89 % of individuals living in LTC facilities had musculoskeletal or neurological conditions (versus 49 and 17 % in HC, respectively; *p* < 0.001). Dementia other than Alzheimer’s disease accounted for the majority of neurological conditions, 74 % of neurological conditions in LTC and 46 % of neurological conditions in HC. In terms of HRQoL, pre-fracture HUI-2 utility scores were also lower in the LTC cohort (0.420 versus 0.560, *p* < 0.001). Statistically significant differences in the percentage composition of fractures were observed between the two groups with, for example, individuals in LTC having more hip fractures (49 % of total versus 29 %).

**Table 1:** Baseline demographics

	Home care (HC)	Long-term care (LTC)	*p*-value (HC vs LTC)
Patients, *n*	3303	1754	
Age, years, mean (SD)	80.6 (10.4)	84.6 (82.7 (8.8)	<0.001
Men, %	19 %	18 %	0.322
Number of medications, mean (SD)	7.3 (2.3)	8.7 (4.9)	<0.001
Number of comorbidities, mean (SD)	2.4 (2.5)	8.0 (2.9)	<0.001
Concurrent medical conditions, %:			
• Endocrine or metabolism	19 %	43 %	<0.001
• Heart or circulation	39 %	77 %	<0.001
• Musculoskeletal	49 %	88 %	<0.001
• Neurological	17 %	89 %	<0.001
• Psychiatric or mood	9 %	73 %	<0.001
• Pulmonary	10 %	59 %	<0.001
• Sensory	11 %	20 %	<0.001
Pre-fracture HUI-2 utility score, mean (SD)	0.560 (0.191)	0.420 (0.004)	<0.001
Fractures, *n*	3303	1754	
Hip, *n* (%)	971 (29 %)	863 (49 %)	<0.001
Wrist, *n* (%)	516 (16 %)	245 (14 %)	
Vertebral, *n* (%)	302 (9 %)	56 (3 %)	
Humerus, *n* (%)	286 (9 %)	113 (6 %)	
Other, *n* (%)	1132 (34 %)	431 (25 %)	
Multiple, *n* (%)	96 (3 %)	46 (3 %)	

*HC* home care, *LTC* long-term care, *SD* standard deviation, *HUI*-*2* Health Utility Index Version 2

### Disutilities associated with fractures

**Fig. 2:**
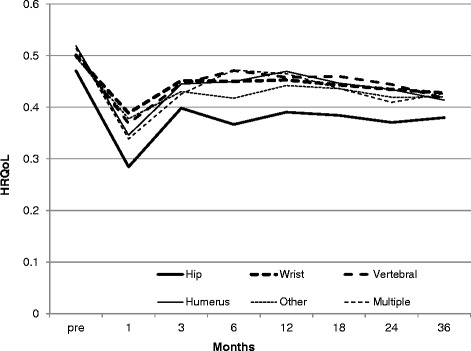
Mean Health Related Quality of Life (HRQoL) Pre- and Post-Fracture, by Type of Fracture (Home Care and Long-term Care cohorts combined)

**Fig. 3:**
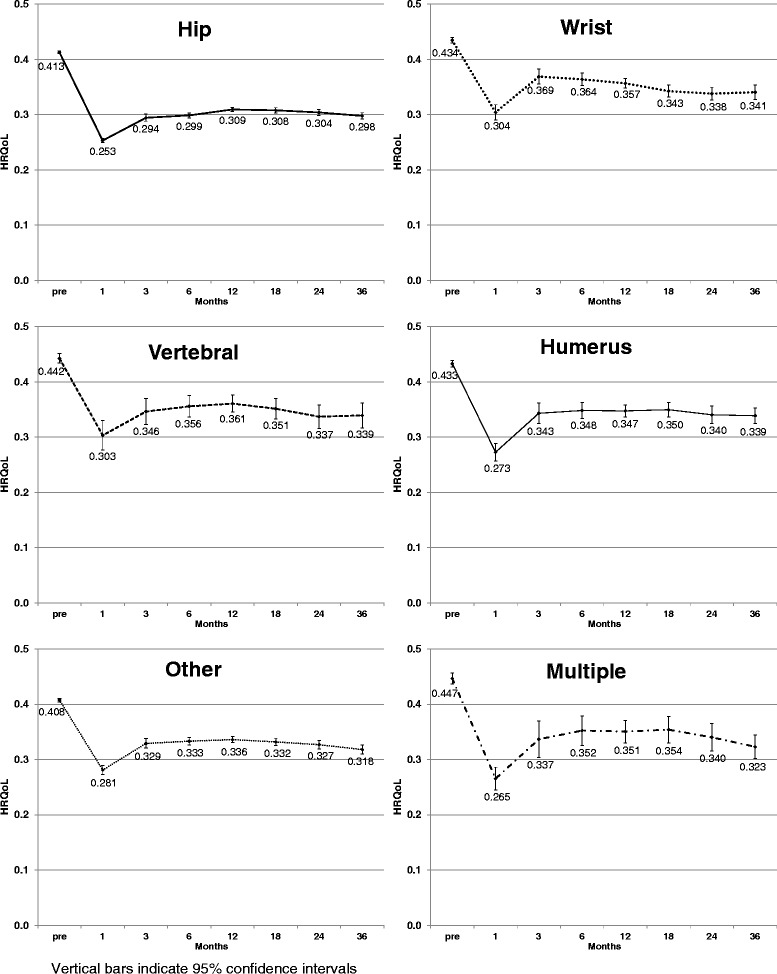
Mean Health Related Quality of Life (HRQoL) Pre- and Post-Fracture for Hip, Wrist, Vertebral, Humerus, Other, and Multiple Fractures (Home Care and Long-term Care cohorts combined)

Figure 2 presents the unadjusted HUI-2 utility scores over time from pre-fracture to 36 months post-fracture by type of fracture for the entire study population (HC and LTC cohorts combined). The overall pattern shows a drop in HRQoL immediately following the fracture and a rebound after the first month. However, for all types of fractures, HRQoL never return to pre-fracture levels in the 3 year post-fracture period (Fig. 2). Among the various types of fractures, individuals who experienced a hip fracture had the lowest pre- and post-fracture HUI-2 utility scores. Figure 3 presents more detailed information on mean HRQoL values and their associated confidence intervals for each time point associated with each type of fracture.

The same patterns in HRQoL following a fracture were observed between the HC and LTC cohorts (Table 2). For both cohorts, and independently of the type of fracture, statistically significant decreases in HUI-2 utility scores were observed 36 months post fracture. All these differences were also clinically meaningful as they exceeded the MCID for the HUI-2 of 0.03. On a scale of 0 to 1, the 3-year disutility varied from −0.089 (other fractures) to −0.138 (humerus) in the HC setting and from −0.097 (humerus) to −0.114 (vertebral) in the LTC setting. There were no statistical or clinical differences between the fractures within each setting (i.e. HC or LTC). Despite baseline differences between patients receiving HC services or living in LTC facilities (i.e., age, comorbidities, pre-level HRQoL), the change in HRQoL 36 months following a fracture was similar between the two cohorts for each type of fracture.

**Table 2:** Unadjusted mean change in HUI-2 utility score 3 years following fracture compared to pre-fracture level

	Home care (HC)		Long-term care (LTC)		HC minus LTC	
	Mean change	SD	Mean change	SD	Difference	*P* value
Hip	−0.109*	0.209	−0.113*	0.146	0.004	0.679
Wrist	−0.095*	0.183	−0.099*	0.146	0.004	0.783
Vertebral	−0.104*	0.198	−0.114*	0.120	0.010	0.655
Humerus	−0.133*	0.182	−0.097*	0.139	−0.036	0.078
Other	−0.089*	0.184	−0.098*	0.140	0.009	0.343
Multiple	−0.103*	0.190	−0.103*	0.148	0.000	1.000

*HC* home care, *LTC* long-term care, *HUI*-*2* Health Utility Index Version 2, *SD* Standard Deviation

*All differences statistically different than zero, *p* < 0.001

To gain a better understanding of the impact of hip and non-hip fractures on each of the 6 domains of the HUI-2 for the HC and LTC cohorts, Table 3 presents the pre-fracture and 36-month post-fracture scores for each domain of the HUI-2. Overall HUI-2 utility scores pre-fracture and 36-month post-fracture are also provided in this table for reference. The domain that provided the largest change in HRQoL following hip or non-hip fractures was related to mobility. There were no clinically meaningful differences in change in HRQoL 3 years following the fracture between hip (−0.109) and non-hip fractures (−0.099). These findings were confirmed by regression analyses which indicated that when adjusting for age, sex, living status (LTC or HC), and comorbidities, having a hip fracture (versus non-hip fracture) was not a statistically significant variable in explaining the change in HRQoL 3 years following a fracture.

**Table 3:** Unadjusted mean change in HUI-2 utility score and domains, hip and non-hip fractures, by residency status

	Home care			Long-term care		
	Pre-fracture	36-month post-fracture	Difference	Pre Fracture	36-month Post-fracture	Difference
Hip fractures						
HUI-2 utility score	0.563	0.454	−0.109*	0.411	0.298	−0.113*
Sensation	0.938	0.911	−0.027*	0.894	0.845	−0.049*
Mobility	0.838	0.768	−0.070*	0.768	0.649	−0.119*
Emotion	0.964	0.958	−0.006	0.927	0.934	−0.007
Cognition	0.976	0.942	−0.034*	0.869	0.812	−0.058*
Self-care	0.901	0.854	−0.047*	0.813	0.803	−0.010*
Pain	0.864	0.872	0.007	0.964	0.971	0.007
Non-hip fracture						
HUI-2 utility score	0.559	0.460	−0.099*	0.426	0.326	−0.100*
Sensation	0.947	0.920	−0.027	0.901	0.868	−0.033
Mobility	0.844	0.790	−0.054*	0.766	0.669	−0.097*
Emotion	0.959	0.951	−0.008*	0.929	0.927	−0.003*
Cognition	0.979	0.952	−0.027*	0.892	0.843	−0.049*
Self-care	0.903	0.862	−0.042*	0.817	0.803	−0.014*
Pain	0.850	0.850	−0.001	0.956	0.964	0.008

*HUI*-*2* Health Utility Index Version 2

*statistically significant, *p* < 0.05

Table 4 presents the results of the regression analyses exploring the impact of covariates on the utility decrement for different populations. In this table, the intercept represents the decrement in utility (3-year post fracture utility minus pre-fracture utility) after adjusting for covariates. Compared to the other populations, individuals with neurological conditions experienced the largest decrement (−0.165 compared to approximately −0.010 for the other populations). When examining the impact of the covariates across the 5 populations presented in this table, age is always a statistically significant variable in explaining the change in HRQoL 3 years following a fracture, while having a hip fracture is only a statistically significant variable for those individuals with neurological conditions. Other variables that have a statistically significant impact on the utility decrement are the presence of musculoskeletal conditions (except for patients with neurological conditions), the presence of neurological conditions (except for residents of LTC), and the presence of a sensory condition for residents of LTC. It should be noted that in this table a negative coefficient indicates a larger decrement while a positive coefficient indicates a smaller decrement in HRQoL.

**Table 4:** Regression analysis to explain change in HUI-2 utility score (36-month post fracture minus pre-fracture)

	HC and LTC combined		HC		LTC		With neurological conditions (HC and LTC)		Without neurological conditions (HC and LTC)	
	Coefficient (SE)	*p*-value	Coefficient (SE)	*p*-value	Coefficient (SE)	*p*-value	Coefficient (SE)	*p*-value	Coefficient (SE)	*p*-value
Intercept*	−0.098 (0.005)	<0.001	−0.098 (0.005)	<0.001	−0.103 (0.018)	<0.001	−0.165 (0.011)	<0.001	−0.100 (0.005)	<0.001
Age (years >80)	−0.002 (0.000)	<0.001	−0.002 (0.000)	<0.001	−0.001 (0.000)	0.004	−0.002 (0.000)	0.000	−0.002 (0.000)	<0.001
Men (vs women)	−0.011 (0.006)	0.084	−0.006 (0.008)	0.472	−0.019 (0.009)	0.042	−0.015 (0.009)	0.094	−0.006 (0.009)	0.465
Hip (vs non-hip)	−0.007 (0.006)	0.233	−0.004 (0.008)	0.632	−0.011 (0.007)	0.131	−0.020 (0.007)	0.007	0.006 (0.008)	0.493
Long term care (vs home care)	0.040 (0.009)	<0.001	–	–	–	–	0.057 (0.010)	<0.001	−0.006 (0.018)	0.736
Endocrine/metabolism condition	−0.003 (0.006)	0.578	−0.005 (0.009)	0.609	−0.001 (0.007)	0.835	−0.002 (0.008)	0.782	−0.006 (0.010)	0.559
Heart/circulation condition	0.008 (0.006)	0.159	0.013 (0.008)	0.110	0.004 (0.009)	0.636	0.009 (0.008)	0.246	0.010 (0.009)	0.249
Musculoskeletal condition	0.019 (0.006)	<0.002	0.021 (0.007)	0.005	0.017 (0.011)	0.123	0.018 (0.010)	0.056	0.021 (0.008)	0.007
Neurological condition	−0.056 (0.007)	<0.001	−0.073 (0.009)	<0.001	−0.007 (0.011)	0.532	–	–	–	–
Psychiatric/mood condition	−0.000 (0.007)	0.919	0.000 (0.011)	0.998	0.001 (0.007)	0.914	0.003 (0.008)	0.724	−0.005 (0.012)	0.663
Pulmonary condition	−0.001 (0.007)	0.934	−0.003 (0.011)	0.774	0.005 (0.009)	0.558	0.003 (0.010)	0.723	−0.002 (0.011)	0.825
Sensory condition	0.009 (0.007)	0.184	−0.001 (0.011)	0.952	0.015 (0.007)	0.040	0.012 (0.008)	0.147	0.006 (0.012)	0.597
Other condition	−0.010 (0.008)	0.156	−0.011 (0.011)	0.337	−0.010 (0.008)	0.224	−0.013 (0.009)	0.131	−0.006 (0.012)	0.620

*Intercept reflects change in HRQoL for age 80 women, and where covariates are set to zero: i.e., non-hip fracture, in home care, without comorbid conditions

For example, the mean change in HRQoL for a group of men aged 85 years in LTC for hip fractures with heart/circulation conditions would be (−0.098) + (−0.002) ∗ 5 + (−0.011) + (−0.007) + (0.040) + (0.008) = − 0.078

*HUI*-*2* Health Utility Index Version 2

When the hip fracture data were analyzed by different types of fractures, the pattern of HRQoL and adjusted decrements in HRQoL at 36 months were similar for fractures of the neck of the femur (*N* = 55 % of all hip fractures) and pertrochanteric fractures (*N* = 43 % of all hip fractures) (i.e. decrements of −0.100 and −0.113, respectively). However, the HRQoL decrement was not significant for subtrochanteric fractures (2 % of all hip fractures) and the overall pattern was not consistent with the two other types of fracture. It should be noted these results were limited due to small sample size of this population (*N* = 44).

### Difference in HRQoL between surviving and deceased individuals

**Fig. 4:**
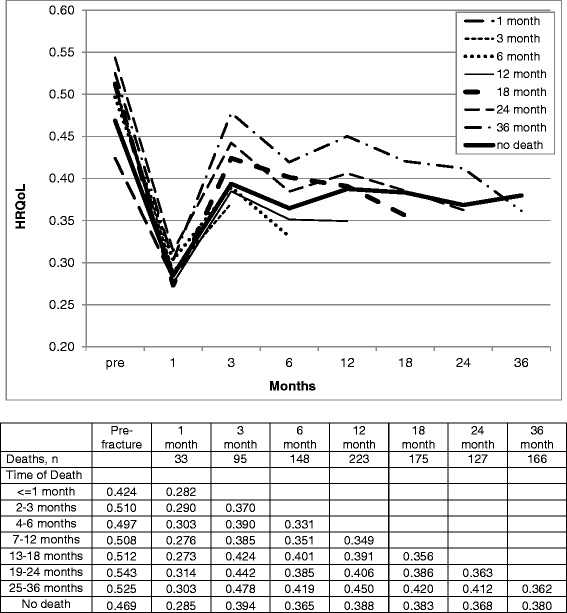
Health Related Quality of Life (HRQoL) Pre- and Post-Fracture, Hip Fracture, by duration of follow-up censored by death (Home Care and Long-term Care cohorts combined)

Our analyses excluded 967 individuals who died within 36 months following a fracture. For those individuals dying within 36 months after a fracture, the decrement in HRQoL post fracture was overall similar to patients who did not die (Fig. 4). In general, there was a clinically important decrease in HRQoL in the period prior to death for all patients independent of the time of death in the range of 0.33 to 0.37 for all lengths of follow-up.

## Discussion

To our knowledge, this is the first time that HRQoL up to 3 years after a fracture has been documented in a large contemporaneous elderly population living in LTC or receiving HC services prior to experiencing a fracture. This is also the largest Canadian study of HRQoL for low-trauma fractures to date. The results of this study are important as they show, in contrast to other studies [11–13], that despite a rebound one month following fracture, there was a long-term permanent decrease in HRQoL for all types of fractures i.e., humerus, vertebral, wrist, multiple and other.

It is somewhat difficult to compare our findings with results from most previous studies [7, 9–11, 27–32] due to the differences in instruments used to measure HRQoL (e.g. EQ-5D, SF-36), study demographics (e.g. younger population) or settings (e.g. community). However, there are several Canadian studies of particular interest as they have used the HUI-2 instrument to measure HRQoL in similar settings. First, the baseline pre-fracture HUI-2 utility scores for our HC study population (e.g. 0.540) were consistent with the HUI-2 utility score reported in a study of Canadian and American older frail adults receiving HC services (0.49) [33]. Another Canadian study reported that the HUI-2 utility score for Canadians aged 80 to 84 and receiving HC services was 0.580 compared to 0.540 for our overall HC [25]. The same study also reported that the HUI-2 utility scores for Canadians aged 80 to 84 living in an institution was 0.33 (compared to 0.39 for our LTC cohort).

A Canadian study of particular interest is the Canadian Multicentre Osteoporosis Study (CaMos) which also used the HUI-2 instrument to document the 5-year impact of fracture on HRQoL. Out of a cohort of approximately 6600 non-institutionalized individuals (mean age: 66 years; 83 % women), 726 experienced a fracture [12]. When compared to individuals who did not experienced a fracture, the 5-year disutilities associated with a hip fracture among women and men were −0.12 and −0.09, respectively (*p* < 0.05), which is very similar to our findings when comparing pre- and post-fracture HUI-2 utility scores. This is an important result as it has been argued that HRQoL losses in institutionalized adults may be lower compared to community dwelling individuals due to their older age and worse baseline health status [15]. CaMos findings also indicated a return to pre-fracture HRQoL for non-hip non-vertebral fractures, which was also reported in other studies at 1 year [11], 2 years [16] or 7 years post fracture [13]. In both the CaMos study and our study, mobility was the HRQoL domain that was most impacted by a fracture.

Our study has several strengths including the large sample size and the 3-year follow-up. This was made possible by linking several administrative databases to identify our study population. While many Canadian administrative databases do not contain information on HRQoL, every Ontario resident receiving nurse-based HC services prescribed by a physician or living in LTC are required to complete the MDS questionnaire during face to face interviews with a nurse or social worker. Since the HUI-2 instrument, a validated HRQoL instrument, is part of the outcomes measured in this questionnaire, the data linkage allows the identification of individuals with fractures (through the hospitalizations or emergency visit databases) living in LTC facilities or receiving HC services. An additional strength of this study is to include all individuals receiving HC services or living in LTC facilities and who had a fracture in the largest province of Canada. In addition to the comprehensiveness in the patient selection, the HUI-2 has shown high reliability and validity [24].

Some of the limitations of the study are related to the nature of the MDS, which is either conducted at home or in LTC facilities by nurses of social workers as part of their normal routine to monitor patient’s progress. As such, the study population excluded individuals who received care in the first few months after fracture in the acute care and non-acute institutional care setting (e.g. admissions to rehabilitation hospital and complex continuing care hospitals). Similarly, to document change in HRQoL pre and post fractures, we excluded anyone who did not receive HC services or live in LTC facilities before the fracture. Although everyone in our study had to have a HUI-2 utility score pre fracture and 3 years after a fracture, the frequency of HUI-2 measurements over time varied, as the MDS is not consistently administered (e.g. every 3 months). For example, the mean number of pre- and post-fracture HRQoL assessments was less than 3 and 6, respectively. For these reasons, we took an average of the HUI-2 utility scores over the year preceding the fracture rather than only using the closest pre-fracture HUI-2 utility score. Since we did not impute the missing data, we did not use time series techniques or repeated measures analyses when estimating losses in HRQoL over time. Rather, we explored changes in HRQoL between two time points for which we had complete data (3-years post fracture minus pre-fracture levels). Due to the recent implementation of the MDS in Ontario, we were only able to follow up patients for 3 years. Future research will allow us to follow the cohort for longer periods as the mandatory data capture of the MDS continues. Another limitation related to the MDS may be related to the capture and reporting of comorbidities, which may be underreported. It is currently unknown, for example, how the MDS reporting of comorbidities compares to claims data. We also did not have access to data on factors other than medical conditions which may have impacted HRQoL, such as level of education, or family and other social support. In addition, the study used a pre-post design to evaluate the impact of low-trauma fractures on HRQoL. As such, we were not able to evaluate the HRQoL of a comparable cohort with no fracture. It should also be noted that the HUI is a general QoL instrument which may not be responsive to small changes in clinical status compared to a disease specific QoL questionnaire. While we believe that the vast majority of the low-trauma fractures included in our analysis were fragility fractures, mechanism of injury cannot be reliably ascertained from the use of ICD-10 codes. As a reference, fragility fracture has been defined as a fracture that occurs spontaneously or following a minor trauma, such as a fall from standing height or less [1]. For these reasons, we concentrated our analyses by identifying through ICD-10 codes all fractures that were not high-trauma fractures. Similarly, while we believe that most of our population was frail due to the high presence of comorbidities, we could not ascertain that all individuals receiving home care services were frail. Finally, while the data are from Ontario, we believe that the results are applicable to the other provinces of Canada.

## Conclusions

In conclusion, the new data generated by this study demonstrate the significant decline in HRQoL associated with low-trauma fractures in an older population in LTC facilities or receiving HC services. This study also demonstrates the long term burden of fracture in the permanent decrement in HRQoL associated with all types of fractures.

### Ethics approval

The data used in the analyses are from the Canadian Institute for Health Information (CIHI). CIHI is a prescribed entity for the purposes of section 45(1) of Ontario’s Personal Health Information Protection Act, 2004 (S.O. 2004, c.3, Schedule A) [34, 35]. This designation allows “health information custodians in Ontario such as hospitals, long-term care facilities and other organizations” to “disclose personal health information to CIHI without the consent of the individuals concerned for the purposes of analysis or compiling statistical information for the planning and management of the health system” [35]. Ethics approval was not required by CIHI for releasing these de-identified secondary data sets provided by CIHI.

### Availability of supporting data

The data used in these analyses were disclosed by CIHI to Authorized Persons under the terms of a Data Non-Disclosure/Confidentiality Agreement and in accordance CIHI privacy policies [36]. Specifically, the data may not be shared with any third party not listed in the Agreement without the prior written authorization of CIHI.

## Additional file

Additional file 1: List of ICD-10 CA codes by type of fracture. (PDF 99 kb)

## Abbreviations

CIHI: Canadian Institute for Health Information
FY: fiscal year
HC: home care
HRQoL: health related quality of life
HUI: Health Utility Index
LTC: long-term care
