# Supplementary material for: Loss of health related quality of life following low-trauma fractures in the elderly
Source: BMC Geriatr. 2016 Apr 19;16:84. doi: 10.1186/s12877-016-0259-5 (PMC4837505; doi:10.1186/s12877-016-0259-5)
Supplement: Additional file 1: — List of ICD-10 CA codes by type of fracture. (PDF 99 kb) [file 12877_2016_259_MOESM1_ESM.pdf]

Additional file 1: List of ICD-10 CA codes by type of fracture

**Fracture type**	**ICD-10 codes relating to fracture type**
Hip	S72.0, S72.1, S72.2
Humerus	S42.2
Vertebral	S22.0, S22.1, S32.0
Wrist	S52 with CCI codes
Other sites:	
• Femur	S72.3, S72.4, S72.7 S72.8, S72.9
• Lower leg (tibia, fibula, ankle, knee, foot)	S82.0-S82.9 (excludes ankle), S92
• Lower arm (radius, ulna)	S52 *unless wrist above*
• Shoulder, upper arm	S42.0-S42.9 *except S42.2*
• Ribs/sternum	S22.2, S22.3, S22.4, S22.8, S22.9
• Lower spine, pelvis	S32.1, S32.3, S32.4, S32.5, S32.7, S32.8
Multiple fractures	T02.1 - T02.9 (*or more than 1 of above*)
